# Economic Development and Forest Cover: Evidence from Satellite Data

**DOI:** 10.1038/srep40678

**Published:** 2017-01-16

**Authors:** Jesús Crespo Cuaresma, Olha Danylo, Steffen Fritz, Ian McCallum, Michael Obersteiner, Linda See, Brian Walsh

**Affiliations:** 1 Vienna University of Economics and Business, Welthandeslplatz 1 1, 1020 Vienna, Austria; 2 Wittgenstein Centre for Demography and Human Capital, Welthandelsplatz 2 2, 1020 Vienna, Austria; 3 International Institute for Applied Systems Analysis, Schlossplatz 1, A-2361 Laxenburg, Austria; 4 Austrian Institute of Economic Research, Arsenal 20, 1030 Vienna, Austria

## Abstract

Ongoing deforestation is a pressing, global environmental issue with direct impacts on climate change, carbon emissions, and biodiversity. There is an intuitive link between economic development and overexploitation of natural resources including forests, but this relationship has proven difficult to establish empirically due to both inadequate data and convoluting geo-climactic factors. In this analysis, we use satellite data on forest cover along national borders in order to study the determinants of deforestation differences across countries. Controlling for trans-border geo-climactic differences, we find that income per capita is the most robust determinant of differences in cross-border forest cover. We show that the marginal effect of per capita income growth on forest cover is strongest at the earliest stages of economic development, and weakens in more advanced economies, presenting some of the strongest evidence to date for the existence of at least half of an environmental Kuznets curve for deforestation.

Substantial increases in human activities over the last century have resulted in forest decline, particularly in the tropical areas of the world. Forest decline manifests as both deforestation—that is, depletion of the tree crown cover to less than 10 percent—and degradation, or negative structural or functional changes that reduce forest quality (e.g. through over-exploitation, repeated fires, or disease)^1^,^2^. Some of the key research in this area has focused on the precise assessment of deforestation rates^3^,^4^, while another central challenge has been to understand the proximate and underlying drivers of deforestation^5^,^6^,^7^. Some of the causes put forth in the literature include increases in overall population^8^,^9^ and specifically in urban areas^10^,^11^, agricultural practices such as shifting cultivation^12^, transport costs and government policies^13^, and agricultural trade^10^.

Empirical support for a hypothetical environmental Kuznets curve for deforestation has until now proven elusive, with studies finding evidence for and against its existence depending on the dataset, estimation method and sample used^14^,^15^,^16^,^17^. The accounting and reporting errors that frequently plague forest statistics further convolute the results of these studies^18^. In this analysis, we use a satellite-based dataset of forest cover and the discontinuities created by national borders as a natural experiment to provide evidence on the relationship between economic development and forest cover^19^ across national borders worldwide for the year 2005. Exploiting discontinuities at national borders is a useful instrument which has been often used in the social sciences to infer causal effects of socioeconomic variables on political and institutional outcomes. Such an approach has been used, for example, to measure the effect of institutions on economic development^20^ or to assess the role of policy measures on sociopolitical outcomes^21^. The use of jurisdictional borders to infer the causal effect of policies is not without criticism^22^. In our setting, lack of comparability of the two sides of the border due to differences in terrain may invalidate inference concerning the drivers of forest cover variation across countries. In addition, the fact that we aim at explaining forest cover around national borders with aggregate, country-wide measures of socioeconomic variables implies that our model assumes that these are also reasonable measures for the differences found in the border. In order to enhance the experimental nature of our design, we use a Homogeneous Response Units (HRU) layer^23^ to ensure comparability of geo-climatic characteristics across countries. These sources allow us to construct a measure of relative forest cover for each pair of neighboring countries, using a buffer of 50 kilometers on both sides of each national border.

Our approach results in a dataset that allows us to identify country-specific socioeconomic determinants of differences in forest cover across countries while keeping environmental factors as constant as possible. Although we do not use data on forest cover change over time, the fact that we employ information on forest cover differences across pairs of countries which share HRUs implies that national borders can be treated as a natural experiment to measure the effect of economic development on forest cover depletion. We estimate regression models for the global sample covering all borders of the world for which data are available. Forest cover differences are assumed to depend on the relative income per capita of the countries on both sides of the border, their growth rate of income per capita, population growth, and rural population density^9^. We include in our specification the difference in squared income per capita levels in order to test for a U-shape relationship between the level of development of a country and forest cover at the border and also entertain threshold regressions in order to allow for nonlinearities in the deforestation Kuznets curve. Our results support the existence of a leveling out of the relationship between forest cover and income per capita with a turning point located at a per capita income level of roughly 5,500 PPP-adjusted 2005 international dollars. This turning point corresponds approximately to the per capita income of Guatemala. We perform a series of robustness checks to ensure that the results found are not driven by particular characteristics of our research design.

## Measuring Forest cover Across National Borders

**Figure 1:**
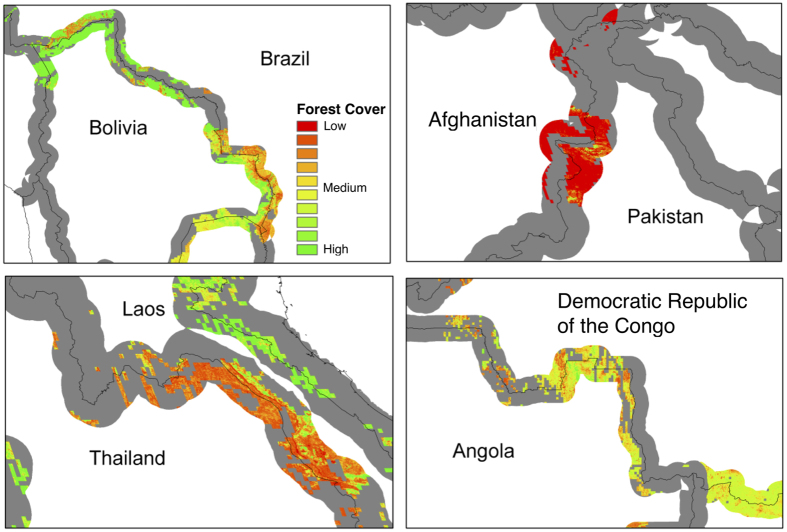
Detail of forest cover along identical homogeneous response units (HRUs). Clockwise from top left: forest cover shown in detail along Bolivia-Brazil, Afghanistan-Pakistan, Angola-Democratic Republic of Congo, and Laos-Thailand-Vietnam borders. Map generated with ArcGIS (v.9.3.1) www.esri.com.

**Figure 2:**
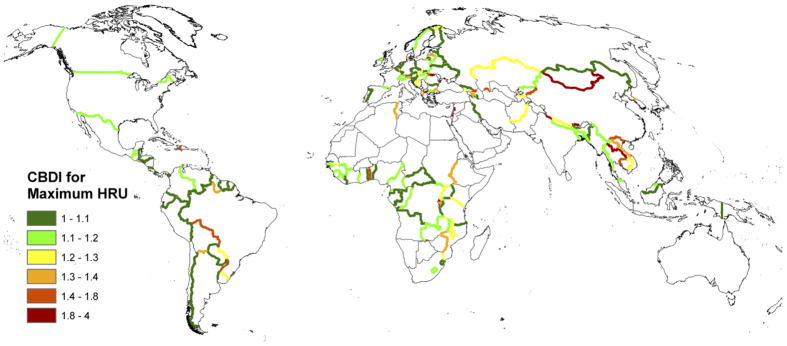
Cross-Border Deforestation Index (CBDI). The index is calculated along all national borders for which data are available. Map generated with ArcGIS (v.9.3.1) www.esri.com.

National borders play the role of a natural experiment in our assessment of the determinants of forest cover differences across countries of the world. Once differences in altitude, slope and soil composition between the two sides of a border are taken into account via HRUs, our identification strategy relies on the fact that differences in forest cover between the two countries on opposite sides of any border are determined by differences in socioeconomic and institutional characteristics between the two nations. We combine forest cover data^19^ with HRUs, which are defined based on classifications of altitude (five classes: 0–300 m, 300–600 m, 600–1100 m, 1100–2500 m and more than 2500 m), slope (seven classes: 0–3°, 3–6°, 6–10°, 10–15°, 15–30°, 30–50° and more than 50°) and soil composition (five classes: sandy, loamy, clay, stony and peat). By concentrating our analysis on forest cover data corresponding to HRUs that cover neighbouring nations, we ensure that the observed variation across country pairs is not driven by differences in altitude, slope or soil composition. Figure 1 presents the forest cover estimates based on HRUs along a 50 km buffer on both sides of four selected borders: Brazil-Bolivia, Afghanistan-Pakistan, Laos-Thailand and Angola-Democratic Republic of Congo. In order to grasp the differences in forest cover existing across borders worldwide, Fig. 2 presents the ratio of forest cover for the HRU with the largest area on both sides of the border, which we label the Cross-Border Deforestation Index (CBDI). In order to ensure that the forest cover difference is not driven by small areas, the CBDI is obtained using the maximum area of HRU shared by bordering countries, requiring that a minimum of 500 km^2^ of the HRU area is present on each side of the border and that at least one of the two sides of the border contains a minimum forest coverage of 20%. See the Methods section for more details on the remote sensing methods employed and a comparison to a similar analysis^24^.

In Fig. 2, borders without color correspond to terrain where the forest cover is less than 20% (e.g. deserts), or where the conditions for computing the CBDI were not met (i.e. the cross-border maximal HRU area is too small). The map shows high values of the index in most continents. For example, strong differences in forest cover between Haiti and the Dominican Republic are picked up very clearly by the method. Large vegetation differentials are also observable between Belize and Guatemala, El Salvador and its neighboring countries, and Brazil and its southern neighbors. Similar differences are observed in Africa: for instance, between Sudan and Ethiopia and between Burundi and both Rwanda and the Democratic Republic of Congo. In Asia, stark cross-border differences in forest cover are observable in particular between China and many of its neighboring nations.

## The Empirical Determinants of Forest Cover: Is There a Deforestation Kuznets Curve?

We start with a simple econometric specification where forest cover in country *i (FC*_*i*_) is assumed to be affected by its level of income per capita (*y*_*i*_), the growth rate of income per capita (Δ*y*_*i*_), population growth (*n*_*i*_) and rural population density (*r*_*i*_)^9^. The relationship between income per capita and forest cover is expected to be U-shaped, since at earlier stages of development the demand for fuelwood is likely to increase with income, while this use of energy is of lesser importance at higher levels of development. Thus, we also include the square of (log) income per capita in our regression^9^. We assume further that forest cover depends on observable and unobservable geo-climatic variables, which are summarized in a vector **z**_*i*_ and linked to the dependent variable by the parameter vector *γ*. The functional form of the modeling exercise is thus given by


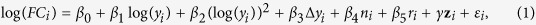


where *ε*_*i*_ the standard disturbance term, is assumed independent and homoscedastic.

Assuming that the data generation process for forest cover in the countries of our sample can be represented by equation (1), cross-border log-differences in forest cover (i.e., the log of our cross-border deforestation index, *CBDI*) can be explained using differences in the explanatory variables in the specification above,


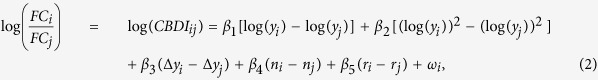


where *ω*_*i*_ is the corresponding error term.

With this construction, *CBDI* computations are based on HRUs, thus ensuring comparability across countries in terms of altitude, slope and soil composition. This implies that the variables in **z** can be considered identical for each one of the pairs and thus play no role in the model based on bilateral cross-border forest cover. Both the *CBDI* value and all explanatory variables not measured in differences of growth rates are evaluated in 2005. We use 2005 as a base year to avoid the potential distortions in GDP and GDP growth data that may have been caused by the global financial crisis. The growth rate differences for income and population refer to the period 2000–2005. Income per capita is measured in PPP-adjusted 2005 international dollars and rural density is measured as total rural population in thousands divided by area^25^.

The first column of Table 1 presents the results of the ordinary least squares estimation of our regression model for the full sample. The results indicate that the covariates usually proposed as factors affecting deforestation have a very limited explanatory power. Rather, differences in cross-border forest cover in the global sample appear to be mostly driven by income per capita differences. Indeed, there is evidence for the existence of a U-shaped relationship between income per capita and forest cover. The U-shaped relationship is robust to including continent dummies (cf. column (2) in Table 1) and institutional quality variables as further controls in the model. The coefficients corresponding to differences in the rule of law and corruption indices^26^ are not individually significant (cf. columns 3 and 4) in Table 1). The inclusion of these variables in our model does not change the conclusions concerning the existence of the environmental Kuznets curve for deforestation, at least in terms of a decreasing effect of income per capita on forest cover as economic development levels increase.

In columns (5) and (6) of Table 1, we enlarge our model by including two variables related to the importance of agriculture as a production sector within each country. The first of these variables measures cross-country differences in agricultural land as a percentage of total land, while the second codifies disparities in agricultural raw material exports as a percentage of merchandising exports. The inclusion of either variable does not affect the empirical evidence concerning the existence of the environmental Kuznets curve for deforestation. Further, although almost 20% of the sample used in the baseline model is missing in this regression due to the lack of availability of data on agricultural exports, we conclude from this exercise that agricultural exports appear to be significantly related to deforestation processes. Keeping other determinants of deforestation constant, an increase of one percent in agricultural exports over total merchandising exports tends to be associated to an average decrease in forest cover of 0.3 percent, a result that is in line with other empirical results available in the literature^10^,^27^.

In order to account for the particular characteristics of border regions, we also perform a regression which includes a variable that accounts for infrastructure around the border. In the spirit of the CBDI, we create a variable that measures road density (log) differences on both sides of national borders and include it in our regressions. This variable is computed using the proportion of grid cells identified as road on each side of the border for the year 2005 and constructing the ratio across neighbouring countries. The estimation results for the model including this indicator as an additional regressor are presented in column (7) of Table 1. The relationship found between income levels and vegetation cover is not affected by the inclusion of this covariate and the effect of border infrastructure appears insignificant once income per capita is controlled for in the regression.

The estimates of our baseline model with continent dummies imply that the income level corresponding to minimum forest cover is roughly at a per capita income level of 5,500 int.$, which in our sample corresponds approximately to the per capita income of Guatemala. Parameter estimates indicate that the income difference between the Democratic Republic of Congo (the country in our sample with lowest income per capita) and Guatemala (the turning point in the estimated environmental Kuznets curve) accounts for approximately a 25% decrease in forest cover. On the other hand, the highest income countries in our sample are predicted to have approximately 10% more forest cover on average and ceteris paribus than do countries near the turning point of the environmental Kuznets curve for deforestation. The estimate of our transition threshold is in line with previous results in the literature, particularly estimates based exclusively on comparisons of the significance of forest cover changes^28^.

**Figure 3:**
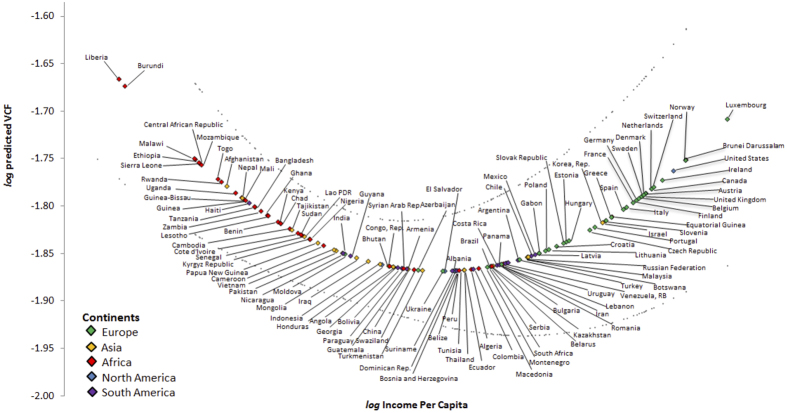
Environmental Kuznetz curve for deforestation. Estimated relationship between income per capita and forest cover.

The fitted environmental Kuznets curve for deforestation, which is implied from the parameter estimates for the baseline model, is depicted in Fig. 3. The parameter estimates for income differences and the difference of squared income obtained from equation (2) correspond to the coefficients of the quadratic relationship between GDP per capita and vegetation cover in ref. 1 and are used to construct the curve. The dispersion of our estimated parameters, combined with the range of observed income values, implies that there is only weak evidence concerning the upward-sloping effect of income on forest cover (i.e. the reforestation part of the environmental Kuznets curve). The heterogeneity within the sample of high income countries included in the analysis may explain the lack of robust evidence for reforestation in this part of the distribution of GDP per capita levels. In addition, contemporary forest policy in emerging markets and highly developed economies has been shaped by the trade-off between reforestation and conservation of biodiversity^29^,^30^. Ecological sustainability arguments have often led to policies in the developed world that aim at the conservation of existing forest stocks instead of the expansion of forest cover^30^.

We perform an additional robustness check by estimating models with a piecewise-linear link between income and forest cover, instead of a quadratic one. This class of models allows for more flexibility in terms of accounting for the asymmetric influence of each country’s level of development on the overall relationship between income and deforestation. We estimate the income threshold that triggers the change in the slope of the deforestation Kuznets curve using the method put forward in Hansen^31^. This exercise results in a threshold estimate of roughly 9200 international dollars, which corresponds to the 64^th^ percentile of our income per capita sample. The estimate of the slope of the relationship between income and forest cover for countries whose income per capita is below the threshold is −0.038 with a standard deviation of 0.02. The estimate for the rest of the sample is −0.026 with a standard deviation of 0.024.

The threshold model thus supports an environmental Kuznets curve for deforestation that lacks a reverting trend for richer economies. To the contrary, the estimation results indicate that the deforestation effect of economic development disappears (but does not revert) as the income level increases. This result is consistent with a similar study that presented evidence of environmental Kuznets curves whose reversal is not significant for other measures of air and water pollution^32^.

We also estimate alternative models using instrumental variables in order to account for potential bilateral causation between forest cover differences and income per capita. We use data on differences in mortality rates of colonial settlers across neighbouring countries as an instrument for income per capita differentials^33^ and obtain estimates of the model parameters using two-stage least squares. Although these models are based on a much smaller sample than that used in the specifications presented in Table 1 (81 observations), the estimates confirm the results presented above (see Supplementary Material). In order to account for potential multicolinearity, we also estimated our specifications using ridge regression methods instead of ordinary least squares. The ridge regression results reinforce the evidence for the existence of a concave relationship between forest cover and income, but weaken the evidence for a negative effect of agricultural exports (see Supplementary Material).

In order to assess the robustness of our results to our definition of the CBDI, we re-estimate our baseline model using two other versions of the index. In particular, we redefine the CBDI based on more stringent conditions concerning the size of the common HRU across the borders used to compute the index. While our baseline CBDI nominally required a minimum of 500 km^2^ of forested areas on each side of the border, we compute two new indices (CBDI_*1000*_ and CBDI_*2500*_) based on alternative minimal HRU area requirements of 1000 and 2500 km^2^, respectively. Results of the estimation of the basic model with continent dummies for each of the new indices are presented in Table 2, together with the original estimates for the *CBDI* based on minimal HRU border coverage of 500 km^2^. The estimates for the alternative measures of cross-border deforestation confirm the existence of the environmental Kuznets curve for deforestation and arrive at similar estimates of the income level corresponding to the turning point in the curve. The use of alternative CBDI definitions therefore does not appear to affect our conclusions regarding the lack of significance of the other determinants in the model. The descriptive statistics for the variables used in the empirical models presented in Tables 1 and 2 are shown in Table 3.

## Conclusions

We make use of the spatial discontinuity provided by national borders in order to assess the socioeconomic determinants of forest cover (and thus deforestation) differences across countries. We combine satellite data on forest cover around national borders with a homogeneous response unit layer that allows us to compare zones of similar geo-climatic characteristics that span national borders. On the basis of this analysis, keeping factors related to climate and terrain differences constant, we observe that countries with a higher GDP per capita tend to have significantly lower forest cover within the group of low-income economies. This phenomenon is not present when we consider the countries with higher levels of income. Thus, our empirical findings provide strong evidence for the existence of at least half of an environmental Kuznets curve for deforestation, which appears to be the most robust factor explaining differences in forest cover across countries once geo-climatic factors are adequately controlled for. This result is also in line with recent evidence based on studies of particular world regions^34^.

Given the fact that economic development is captured through GDP per capita in our analysis, further research is required to understand the particular mechanisms that generate the robust causal relationship between income and forest cover elucidated in this analysis. Because of the high cross-country correlation between GDP per capita and other socioeconomic variables, the environmental Kuznets curve for deforestation may be driven, among other factors, by changes in the yields of non-forested land^10^ as well as by access to credit, which in turn affects the liquidity constraints faced by forest owners in developing economies^35^. The role of agricultural trade as a driver of deforestation, which has also been highlighted in the recent empirical literature^10^,^27^ and which is also found to be a potentially important determinant of forest cover differences across countries in our study, deserves further scrutiny.

## Methods and Technical Appendix

### Homogeneous Response Units (HRU)

In order to ensure consistency in environmental conditions for the terrain across national borders, the Homogenous Response Units (HRU) layer was used^23^. HRUs are defined based on classifications of altitude (five classes: 0–300 m, 300–600 m, 600–1100 m, 1100–2500 m and more than 2500 m), slope (seven classes: 0–3°, 3–6°, 6–10°, 10–15°, 15–30°, 30–50° and more than 50°) and soil composition (five classes: sandy, loamy, clay, stony and peat).

HRU zone-specific altitude, slope or soil class values which have been assigned to 5 minute spatial resolution pixels represent the spatially most frequent class value (not average) taken from the input data. In total, 150 unique combinations of altitude, slope and soil class resulted from the HRU delineation process globally. Each delineated HRU zone is indexed by a numerical code assembled from a code of the altitude, slope and soil at the first, second and third position in the string, respectively. The HRU is a 5 arc minute spatial resolution grid. The full HRU dataset along with metadata is available for download at http://doi.pangaea.de/10.1594/PANGAEA.775369.

### Vegetation Continuous Fields (VCF)

Data on forest cover percent were obtained from the Moderate Resolution Imaging Spectroradiometer (MODIS) on NASA’s Terra spacecraft. The Terra MODIS Vegetation Continuous Fields (VCF) product is a sub-pixel-level representation of surface forest cover estimates globally^36^. Designed to continuously represent Earth’s terrestrial surface as a proportion of basic vegetation traits, it provides a gradation of percent tree cover. The VCF product is generated yearly and produced using monthly composites of Terra MODIS 250 and 500 meters Land Surface Reflectance data, including all seven bands, and Land Surface Temperature. The VCF products are validated to stage-1, which means that their product accuracy was estimated through an assessment of the accuracy using training data and from limited *in situ* field validation datasets. The MODIS continuous fields of forest cover algorithm is described in Hansen *et al*.^19^,^36^.

The output of the algorithm is the percent canopy cover per 500-m MODIS pixel. Here percent canopy refers to the amount of skylight obstructed by tree canopies equal to or greater than 5 m in height and is different than percent crown cover (crown cover = canopy cover + within crown skylight). Using a buffer of 50 km on both sides of each national border, we obtain a measure of relative vegetation continuous field for each pair of neighbouring countries. Although the use of 50 km as a buffer may be considered a limitation of the analysis, the high correlation between estimates of vegetation cover differentials based on buffers of 25 km and 50 km across national borders indicates that this particular choice does not appear to drive the results presented. We assume that the high correlation would also hold true at the HRU level for the 25 km buffer size aalthough we did not condition the regression on HRUs, since they represent more homogenous areas in terms of environmental conditions. Data used in this study were obtained from www.landcover.org, collection 4, version 3, 500 m for the year 2005. The VCF dataset used in this study was compared and found to be highly correlated (>0.9) for the year 2005 with the figures provided by Hansen *et al*.^24^, which are derived from 30 m Landsat data. The 2005 forest cover map was based on tree cover in 2000 and forest loss for years 2000–2005^24^. A sample of about 600,000 random points in border regions (291,903 points are in the tropics – between −23.5 and 23.5 latitude) was created for the correlation analysis. Buffer zones were created for the random points at 250 m. Both forest datasets were resampled to 50 m using mean forest cover in order to compute the correlation.

## Additional Information

**How to cite this article**: Crespo Cuaresma, J. *et al*. Economic Development and Forest Cover: Evidence from Satellite Data. *Sci. Rep.* **7**, 40678; doi: 10.1038/srep40678 (2017).

**Publisher's note:** Springer Nature remains neutral with regard to jurisdictional claims in published maps and institutional affiliations.

## Supplementary Material

Supplementary Material

## Figures and Tables

**Table 1:** Estimation results, determinants of bilateral forest cover differences.

	(1)	(2)	(3)	(4)	(5)	(6)	(7)
Income per capita	−0.441**	−0.448**	−0.469**	−0.473**	−0.352*	−0.700**	−0.670**
	[0.179]	[0.178]	[0.182]	[0.187]	[0.186]	[0.321]	[0.297]
(Income per capita)^2^	0.0256**	0.0261**	0.0281**	0.0283**	0.0202*	0.0400**	0.038**
	[0.0113]	[0.0112]	[0.0116]	[0.0121]	[0.0116]	[0.0193]	[0.0178]
Income growth	0.0484	0.0104	0.000278	−0.00343	0.0225	−0.0672	−0.0659
	[0.0510]	[0.0494]	[0.0492]	[0.0510]	[0.0482]	[0.164]	[0.162]
Population growth	0.259	0.319	0.354	0.333	0.237	−0.11	−0.0279
	[0.609]	[0.573]	[0.568]	[0.573]	[0.587]	[0.710]	[0.716]
Rural pop. density	−0.34	−0.344	−0.322	−0.336	−0.161	0.0106	−0.0029
	[0.327]	[0.344]	[0.342]	[0.344]	[0.389]	[0.466]	[0.460]
Rule of law			−0.0234				
			[0.0240]				
Corruption				−0.0237			
				[0.0260]			
Agricultural land					−0.125		
					[0.0942]		
Agric. raw material exports						−0.377**	−0.344**
						[0.147]	[0.152]
Border road density							0.0238
							[0.0207]
Continent dummies	No	Yes	Yes	Yes	Yes	Yes	Yes
Observations	189	189	189	189	183	154	154
R-squared	0.046	0.077	0.080	0.080	0.083	0.066	0.075

Robust standard errors in bracketsparenthesis. *(**) stands for significance at the 10%(5%) level. Dependent variable is the (log) cross-border deforestation index (CBDI) in 2005. Income per capita refers to the log of GDP per capita in 2005, while income growth is the growth of GDP per capita 2000–2005 based on World Development Indicators 2010 data^25^. Rule of law and corruption indices are sourced from the Heritage Foundation^26^. Number of observations refers to country pairs.

**Table 2:** Estimation results for alternative CBDI definitions.

	*CBDI*	*CBDI*_*1000*_	*CBDI*_*2500*_
Income per cap.	−0.448**	−0.725**	−0.509*
	[0.178]	[0.303]	[0.285]
(Income per cap.)^2^	0.0261**	0.0418**	0.0298*
	[0.0112]	[0.0185]	[0.0179]
Income growth	0.0104	0.0452	0.0559
	[0.0494]	[0.0790]	[0.0756]
Population growth	0.319	0.275	0.387
	[0.573]	[0.847]	[0.811]
Rural pop. density	−0.344	−0.251	−0.413
	[0.344]	[0.512]	[0.443]
Continent dummies	Yes	Yes	Yes
Observations	189	184	177
R-squared	0.077	0.112	0.112

Robust standard errors in parenthesis. *(**) stands for significance at the 10%(5%) level. Dependent variable is the (log) cross-border deforestation index (CBDI) in 2005. Income per capita refers to the log of GDP per capita in 2005, while income growth is the growth of GDP per capita 2000–2005^25^. Number of observations refers to country pairs.

CBDI is nominally calculated for HRUs with at least 500 km^2^ on each side of a national border. In the second and third columns, CBDI correlations are recalculated for HRUs with at least 1000 km^2^ and 2500 km^2^ forested area, respectively, on each side.

**Table 3:** Descriptive statistics for variables used in the regression models.

Variable	Observations	Mean	Std. Dev.	Minimum	Maximum
log(CBDI)	189	0.028	0.286	−0.915	1.387
log(CBDI_1000_)	184	0.077	0.457	−1.561	1.387
log(CBDI_2500_)	177	0.084	0.399	−0.995	1.387
Population growth (country pair differences)	189	−0.002	0.042	−0.130	0.125
Income growth (country pair differences)	189	−0.004	0.284	−1.832	1.918
Income per capita (country pair differences)	189	−0.030	0.863	−2.556	2.609
Rural pop. density (country pair differences)	189	0.002	0.067	−0.265	0.553
Rule of law (country pair differences)	189	0.021	0.808	−1.889	2.731
Corruption (country pair differences)	189	0.045	0.754	−1.827	2.971
Agricultural land (country pair differences)	183	0.011	0.248	−0.661	0.790
Agr. raw material exports (country pair differences)	154	−0.006	0.105	−0.687	0.554
