# Supplementary material for: Economic Development and Forest Cover: Evidence from Satellite Data
Source: Sci Rep. 2017 Jan 16;7:40678. doi: 10.1038/srep40678 (PMC5238442; doi:10.1038/srep40678)
Supplement: Supplementary Material [file srep40678-s1.doc]

**Supplementary Material**

**Economic Development and Forest Cover: Evidence from Satellite Data**

***Jesús Crespo Cuaresma^1,2,3,4^,*, Olha Danylo^3^, Steffen Fritz^3^, Ian McCallum^3^, Michael Obersteiner^3^, Linda See^3^, Brian Walsh^3^***

***Affiliations***

*^1^ Vienna University of Economics and Business, Welthandeslplatz 1 1, 1020 Vienna, Austria.*

*^2^ Wittgenstein Centre for Demography and Human Capital, Welthandelsplatz 2 2, 1020*

*Vienna, Austria.*

*^3^ International Institute for Applied Systems Analysis, Schlossplatz 1, A-2361 Laxenburg,*

*Austria.*

*^4^ Austrian Institute of Economic Research, Arsenal 20, 1030 Vienna, Austria.*

* Corresponding author. Email: jcrespo@wu.ac.at

**Robustness of the results to alternative estimation methods**

Table S1 presents the results of the estimation of specifications of our model using different methods. In the first column of Table S1 we present the results obtained using two-stage least-square estimation, where the income variables have been instrumented making use of colonial settler mortality. Following the results in *(33)*, differences in mortality rates of colonial settlers are considered to create exogenous variation that helps us explain differences in the quality of institutions that countries imported from colonial powers. These differences in institutional quality, in turn, have been shown to robustly affect income per capita differences in present times. Due to the restricted sample of countries for which the settler mortality data are available, the number of observations in our regression is restricted to 81 (compared to 189 in the original regressions). The Kuznets curve for deforestation is still present in the results of the model estimated using instrumental variables (albeit at the 10% significance level), with a steeper deforestation slope than the results obtained using ordinary least squares estimation in the full sample but a similar level of income in the turning point. Such a result is not surprising given the fact that few developed economies (which would tend to fall in the flat or upward sloping of the Kuznets curve for vegetation cover) are included in the subsample used for instrumental variables estimation.[[1]](#footnote-2)

In order to assess the potential effect that multicolinearity may have on the results of the models used in our study (see correlation matrix of the regressors used in our model in Table S2), we reestimated the central specifications making use of ridge regression methods. Ridge regression embodies a penalty on the size of the estimated parameters, thus inducing shrinkage in the estimates and providing benefits in the presence of correlated regressors. The estimates obtained using ridge regression can be found in columns 2 to 5 of Table S1 and support the results obtained using ordinary least square methods concerning the existence of a deforestation Kuznets curve. The evidence concerning the robustness of the effect of agricultural exports on forest cover is however weakened when ridge regression is used as an estimation method. In addition, we also report the estimates of regression models based on single determinants in Table S3, which confirm our findings in the multivariate stetting.

|  | IV | Ridge | Ridge | Ridge | Ridge |
| --- | --- | --- | --- | --- | --- |
| Income per capita | -1.36* | -0.434** | -0.441** | -0.684** | -0.668** |
|  | [0.709] | [0.187] | [0.187] | [0.332] | [0.331] |
| (Income per capita)^2^ | 0.0833* | 0.0252** | 0.0257** | 0.0391** | 0.0379** |
|  | [0.0477] | [0.0113] | [0.0113] | [0.0192] | [0.0191] |
| Income growth | 0.189 | 0.048 | 0.0104 | -0.0675 | -0.0633 |
|  | [0.243] | [0.0744] | [0.0767] | [0.160] | [0.160] |
| Population growth | 0.275 | 0.29 | 0.349 | -0.0736 | -0.0344 |
|  | [1.053] | [0.520] | [0.520] | [0.605] | [0.603] |
| Rural pop. density | -0.374 | -0.331 | -0.333 | 0.0302 | 0.0106 |
|  | [0.430] | [0.308] | [0.307] | [0.393] | [0.391] |
| Agric. raw material exports |  |  |  | -0.00357 | -0.00335 |
|  |  |  |  | [0.00235] | [0.00235] |
| Border road density |  |  |  |  | 0.0236 |
|  |  |  |  |  | [0.0166] |
| Continent dummies | No | No | Yes | Yes | Yes |
| Observations | 81 | 189 | 189 | 199 | 198 |
| Standard errors in brackets.*(**) stands for significance at the 10%(5%) level. Column labelled IV presents the estimation results based on instrumental variable estimations using settler mortality differences across countries with a common border as an instrument for income per capita. Columns labelled Ridge present results based on ridge regression methods. | | | | | |

**Table S1: Results based on alternative estimation methods**

|  | Population growth diff. | GDP growth diff. | Income per capita diff | Income per capita squared diff | Rural density diff. | Agr. Exportts diff | Border road |
| --- | --- | --- | --- | --- | --- | --- | --- |
| Population growth diff. | 1.00 | -0.23 | -0.05 | -0.02 | -0.04 | 0.04 | -0.05 |
| GDP growth diff. | -0.23 | 1.00 | -0.08 | -0.11 | -0.10 | 0.11 | -0.02 |
| Income per capita diff | -0.05 | -0.08 | 1.00 | 0.99 | -0.20 | -0.16 | 0.11 |
| Income per capita squ. diff | -0.02 | -0.11 | 0.99 | 1.00 | -0.18 | -0.16 | 0.11 |
| Rural density diff. | -0.04 | -0.10 | -0.20 | -0.18 | 1.00 | -0.08 | 0.03 |
| Agr. Exportts diff | 0.04 | 0.11 | -0.16 | -0.16 | -0.08 | 1.00 | -0.08 |
| Border road | -0.05 | -0.02 | 0.11 | 0.11 | 0.03 | -0.08 | 1.00 |

**Table S2: Correlation matrix of regressors**

| Income per capita | -0.456*** |  |  |  |  |
| --- | --- | --- | --- | --- | --- |
|  | [0.163] |  |  |  |  |
| (Income per capita)^2^ | 0.0268** |  |  |  |  |
|  | [0.0103] |  |  |  |  |
| Income growth |  | -0.00947 |  |  |  |
|  |  | [0.0536] |  |  |  |
| Population growth |  |  | 0.5780 |  |  |
|  |  |  | [0.526] |  |  |
| Rural pop. density |  |  |  | -0.300 |  |
|  |  |  |  | [0.227] |  |
| Agric. raw material exports |  |  |  |  | -0.00313* |
|  |  |  |  |  | [0.00180] |
| Continent dummies | Yes | Yes | Yes | Yes | Yes |
| Observations | 190 | 190 | 199 | 198 | 155 |
| R-squared | 0.054 | 0.02 | 0.023 | 0.02 | 0.025 |

Robust standard errors in brackets.*(**) stands for significance at the 10%(5%) level

**Table S3: Regression models with individual regressors**

1. The following countries are included in the sample of the model estimated by instrumental variables: Algeria, Angola, Argentina, Bangladesh, Belize, Benin, Bolivia, Brazil, Burundi, Cameroon, Canada, Central African Republic, Chad, Chile, China, Colombia, Congo, Costa Rica, Cote d'Ivoire, Democratic Republic of the Congo, Dominican Republic, Ecuador, El Salvador, Ethiopia, Gabon, Ghana, Guatemala, Guinea, Guinea-Bissau, Guyana, Haiti, Honduras, India, Indonesia, Kenya, Lao People's Democratic Republic, Liberia, Malaysia, Mali, Mexico, Nicaragua, Nigeria, Panama, Papua New Guinea, Paraguay, Peru, Rwanda, Senegal, Sierra Leone, Sudan, Suriname, Thailand, Togo, Tunisia, Uganda, United Republic of Tanzania, United States of America, Uruguay, Venezuela and Vietnam. [↑](#footnote-ref-2)
